# Dietary restriction and fasting downregulate complement activity

**DOI:** 10.1186/1753-6561-6-S3-P66

**Published:** 2012-06-01

**Authors:** Shushimita Shushimita, Pieter van der Pol, Ron WF de Bruin, Jan NM IJzermans, Cees van Kooten, Frank JMF Dor

**Affiliations:** 1 Department of Surgery, Erasmus MC, University Medical Center, Rotterdam, The Netherlands; 2 Department of Nephrology, Leiden University Medical Center, Leiden, The Netherlands

## Background

Seventy-two hours of preoperative fasting (F) or 2 weeks of 30% dietary restriction (DR) offers robust protection against renal ischemia-reperfusion injury (IRI) in mice. However, the mechanism remains to be elucidated. We hypothesize that immunomodulation plays a pivotal role. Innate immunity, especially the complement system, is crucial in the pathophysiology of IRI. Therefore, we investigated the impact of fasting and dietary restriction on complement activation pathways.

## Materials and methods

Male C57BI/6 mice were fed *ad libitum* (AL) or underwent 72 hours fasting or 30% dietary restriction for 2 weeks (n=8/group). Consequently blood was drawn and serum was aliquoted and stored at -80°C. Functional activity of the complement activation pathways (classical (CP), lectin (LP) and alternative pathway (AP)) was assessed by ELISA using immobilized ligands. Deposition of C3 and C9 as a measure of complement activity along with concentration of upstream complement initiating proteins (MBL-A and -C, and C1q) was determined.

## Results

A significant downregulation in CP and LP activity by dietary restriction and in CP, LP and AP activity by fasting was observed, compared to the ad libitum group. The activation of both C3 and C9 in the dietary restriction and fasting group was significantly downregulated (p≤0.002) in CP, LP and AP (except for C3 activation in the AP of the dietary restriction group). The MBL-A concentrations were significantly lower (p≤0.001) after dietary restriction and fasting; 15.4 µg/ml (DR) and 12.4 µg/ml (F) compared to 19.9 µg/ml in ad libitum mice. MBL-C concentrations were also significantly lower (p≤0.0001) in the dietary restriction and fasting groups; 89.4 and 49.5 µg/ml respectively compared to 109.6 µg/ml in the ad libitum group. C1q concentration was only significantly lower in the fasted group (p≤0.0001).

## Conclusion

Dietary interventions downregulate complement activation pathways. Compared to dietary restriction, fasting has a more pronounced effect. CP seems to be more affected by dietary restriction while AP is most affected by fasting. To our knowledge, our data for the first time show that DR and fasting cause downregulation of complement activation pathways. Therefore, we conclude that complement downregulation may be one of the mechanisms by which dietary interventions protect against renal IRI.

